# Protocol for development and validation of a prediction model for 5-year risk of incident overactive bladder in the general population: the Nagahama study

**DOI:** 10.1186/s12894-021-00848-x

**Published:** 2021-05-13

**Authors:** Satoshi Funada, Yan Luo, Takashi Yoshioka, Kazuya Setoh, Yasuharu Tabara, Hiromitsu Negoro, Shusuke Akamatsu, Koji Yoshimura, Fumihiko Matsuda, Toshi A. Furukawa, Orestis Efthimiou, Osamu Ogawa

**Affiliations:** 1 Department of Urology, Faculty of Medicine, Kyoto University Graduate School of Medicine, 54 Shogoinkawahara-cho, Sakyo-ku, Kyoto, 606-8507 Japan; 2 Department of Health Promotion and Human Behavior, Kyoto University School of Public Health, Kyoto, Japan; 3 Center for Innovative Research for Communities and Clinical Excellence (CiRC2LE), Fukushima Medical University, Fukushima City, Fukushima Japan; 4 Department of Healthcare Epidemiology, Kyoto University Graduate School of Medicine and School of Public Health, Kyoto, Japan; 5 Center for Genomic Medicine, Faculty of Medicine, Kyoto University Graduate School of Medicine, Kyoto, Japan; 6 Department of Urology, University of Tsukuba, Ibaraki, Japan; 7 Department of Urology, Shizuoka General Hospital, Shizuoka, Japan; 8 Institute of Social and Preventive Medicine, University of Bern, Bern, Switzerland; 9 Department of Psychiatry, University of Oxford, Oxford, UK

**Keywords:** Urinary bladder, Longitudinal analysis, Cohort study, Risk calculator

## Abstract

**Background:**

An accurate prediction model could identify high-risk subjects of incident Overactive bladder (OAB) among the general population and enable early prevention which may save on the related medical costs. However, no efficient model has been developed for predicting incident OAB. In this study, we will develop a model for predicting the onset of OAB at 5-year in the general population setting.

**Methods:**

Data will be obtained from the Nagahama Cohort Project, a longitudinal, general population cohort study. The baseline characteristics were measured between Nov 28, 2008 and Nov 28, 2010, and follow-up was performed every 5 years. From the total of 9,764 participants (male: 3,208, female: 6,556) at baseline, we will exclude participants who could not attend the follow-up assessment and those who were defined as having OAB at baseline. The outcome will be incident OAB defined using the Overactive Bladder Symptom Score (OABSS) at follow-up assessment. Baseline questionnaires (demographic, health behavior, comorbidities and OABSS) and blood test data will be included as predictors. We will develop a logistic regression model utilizing shrinkage methods (LASSO penalization method). Model performance will be evaluated by discrimination and calibration. Net benefit will be evaluated by decision curve analysis. We will perform an internal validation and a temporal validation of the model. We will develop a web-based application to visualize the prediction model and facilitate its use in clinical practice.

**Discussion:**

This will be the first study to develop a model to predict the incidence of OAB.

## Background

Overactive bladder (OAB) is defined as “a symptom characterized by urinary urgency, with or without urgency incontinence, usually with urinary frequency and nocturia in the absence of infection or other obvious pathology” [1]. The prevalence of OAB is estimated from 10 to 20% and increases with age [2–4]. OAB might significantly decrease the HRQOL in patients [5] and increase the expenditure of medical cost [6]. The prevalence of OAB is increasing in an aging society and the negative impacts on HRQOL and medical cost are becoming even more serious.

Population-based prediction models would be helpful for population health planning and policy decision-making [7]. The same is expected for OAB prediction model because some good behaviors, such as healthy eating habit, keeping healthy weight, quitting smoking and pelvic floor muscle exercise, are efficient for keeping the bladder as healthy as possible [8]. If an accurate prediction model can be developed, high-risk subjects could be identified and encouraged to such good habits at an early stage, which might prevent incident OAB and save on the medical cost related to pharmacotherapy. If such a model could be made freely available to the general public online and encourage good habits for a healthy bladder, it could change user’s behavior to prevent incident OAB and impact on health care providers within clinical practice guidelines to inform decision making in the clinical setting. However, to our knowledge, no model has been developed to predict the new-onset of OAB in the literature. This could be due to lack of sufficient data to develop such a prediction model in terms of sample size, retrospective study design, and/or important predictors. We have recently reported longitudinal analyses of voiding dysfunction using a large prospective cohort data from the general population [9, 10]. These data can be used to develop an adequate model to predict new-onset OAB in the general population.

In this study, we will use a large prospective Japanese general population cohort to develop a model to predict the new-onset OAB at 5-year. We will develop a model consisting of only questionnaires and will compare the performance with another model including blood test. If the performance of the two models is deemed to be comparable, we will choose the model without blood testing, aiming to make the model more easily accessible, even by the general population. As the mechanism of incident OAB could be different between male and female due to factors such as the prostate gland, menopause and delivery, we will develop separate models for each sex. In addition, we will develop a web-based application to visualize the results interactively.

## Methods/design

We will follow the Transparent Reporting of a Multivariable Prediction Model for Individual Prognosis or Diagnosis (TRIPOD) checklist for developing and validating our prediction model [7].

### Study design and source of data

We will use the Nagahama cohort [4, 9, 10], a prospective population-based cohort study in the Nagahama city, a Japanese rural city of 125,000 inhabitants. Recruitment was performed between Nov 28, 2008 and Nov 28, 2010, and the baseline characteristics were measured. Follow-up was performed every 5 years after baseline assessment, and the follow-up assessment was performed between July 28, 2013 and Feb 10, 2016. The cohort study was approved by the ethics committee of Kyoto University Graduate School of Medicine (no. G278) and by the Nagahama Municipal Review Board. Written informed consent was obtained from all participants.

### Study population

**Fig. 1:**
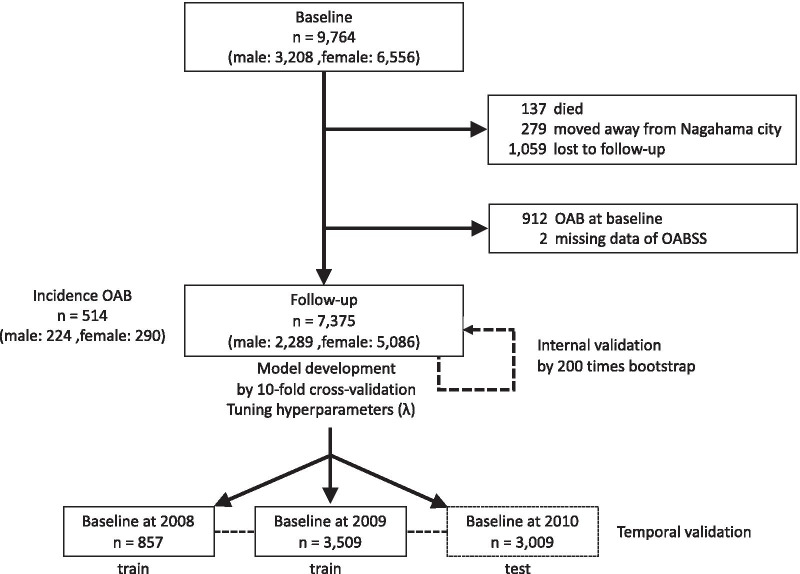
Study flow chart

Participants were recruited from the general community residents of Nagahama city. Inclusion criteria were as follows: age 30–74 years; ability to independently participate in health examinations; no difficulties in communicating in Japanese; no serious diseases, symptoms, or other health issues; and voluntary participation. From the total 9764 participants (male: 3208, female: 6556) at baseline, we will exclude 1475 participants who did not attend the follow-up assessment because of death (n = 137), moving from Nagahama City (n = 279) or some other unknown reason (n = 1059). From the 8289 follow-up participant, we will exclude 912 OAB participant and 2 missing data of OAB at baseline, and 7375 participants (male: 2289, female: 5086) will be used in this analysis. The study flow chart is shown in Fig. 1.

### Study outcome

The outcome will be new-onset OAB at 5-year follow-up assessment. We will use OABSS, a self-report measure assessing of urinary urgency validated by Homma et al. [11]. The questionnaire consists of the following items: (i) How many times do you typically urinate from waking in the morning until sleeping at night? (ii) How many times do you typically wake up to urinate from sleeping at night until waking in the morning? (iii) How often do you have a sudden desire to urinate, which is difficult to defer? (iv) How often do you leak urine because you cannot defer the sudden desire to urinate? OAB will be defined as a total OABSS score ≥ 3, with an urgency score (iii) ≥ 2 [11]. The number of new-onset OAB at follow-up assessment will be 224 for male and 290 for female.

### Sample size calculation

We calculated the minimum sample size needed to build a prediction model using the criteria recommended by Riley et al. [12]. For these calculations it is required to provide an expected R^2^ value. As there has been no prediction model of incident OAB previously, we have set R^2^ = 0.10 as a conservative choice in this study. Based on the number of events in our dataset (224 male, 290 female) and the selected value for R^2^, we calculated the upper limit of the number of predictors to be 27 for the model for males and 35 for females.

### Candidate predictor variables

Based on previous reports [13–18] and expert opinion, we will initially include in the mode the following candidate predictors variables which were measured at baseline; demographic variables (age, body mass index [BMI], delivery, menopause, smoking status, alcohol habit, walking habit), history of comorbidities (hypertension, hyperlipidemia, diabetes, ischemic heart disease, stroke, kidney disease, cancer, depression, sleep disturbance, obstructive sleep apnea [OSA], benign prostate disease and prostate cancer [PCa]), questionnaires specific to OAB (OABSS question 1, question 2, question 3 and question 4) and blood test (HbA1c, B-type natriuretic peptide [BNP], the estimated glomerular filtration rate [eGFR]) and prostate specific antigen [PSA]). Trained physicians and research assistants administered the standardized questionnaire in which participants provided clinical background information, such as lifestyle and medical history. Anthropometric and physiological measurements were taken by trained nurses.

Age will be treated as continuous value. BMI will be calculated as continuous values using height and weight data. Smoking status will be categorized as a dichotomous variable either current or none smoker. Alcohol habit will be categorized as a dichotomous variable either current or none drinker. Walking habit will be categorized as a dichotomous variable by the questionnaire: walking for ≥ 1 h or < 1 h. Delivery will be categorized as a dichotomous variable either experienced or not. History and medical comorbidities (menopause, hypertension, hyperlipidemia, diabetes, ischemic heart disease, stroke, kidney disease, cancer, depression, sleep disturbance, OSA, benign prostate disease, prostate cancer) will be categorized as a dichotomous variable by the questionnaire: yes or no. OABSS question 1, question 2, question 3 and question 4 will be treated as continuous variables. Blood samples (HbA1c, BNP, creatine, PSA) will be used as continuous values. eGFR will be calculated from serum creatinine levels using the following formula: 194 × serum creatinine^−1.094^ × age^−0.287^ (× 0.739 if female).

We will develop two models based on the sample size calculations as follows (Table 1a and b);

- Model 1 including demographic questionnaires (age, BMI, delivery and menopause), health behavior questionnaires (smoking status, alcohol habit and walking habit) and comorbidities questionnaires (hypertension, hyperlipidemia, diabetes, ischemic heart disease, stroke, kidney disease, cancer, depression, sleep disturbance, OSA, prostate disease and prostate cancer) and questionnaires specific OAB (OABSS question 1, question 2, question 3 and question 4)
- Model 2 consisting of Model 1 plus blood test (HbA1c, BNP, eGFR and PSA)

**Table 1:** Candidate predictor variables for new-onset OAB in (a) male, (b) female

Variable	Scale	Number of parameters	
		Model 1	Model 2
(a)			
*Demographic*			
Age	Continuous	1	1
BMI	Continuous	1	1
*Health behavior*			
Smoking status	Dichotomous	1	1
Alcohol habit	Dichotomous	1	1
Walking habit	Dichotomous	1	1
*Comorbidity*			
Hypertension	Dichotomous	1	1
Hyperlipidemia	Dichotomous	1	1
Diabetes	Dichotomous	1	1
Ischemic heart disease	Dichotomous	1	1
Stroke	Dichotomous	1	1
Kidney disease	Dichotomous	1	1
Cancer	Dichotomous	1	1
Depression	Dichotomous	1	1
Sleep disturbance	Dichotomous	1	1
Obstructive sleep apnea	Dichotomous	1	1
Prostate disease	Dichotomous	1	1
Prostate cancer	Dichotomous	1	1
*OABSS*			
Question 1	Continuous	1	1
Question 2	Continuous	1	1
Question 3	Continuous	1	1
Question 4	Continuous	1	1
*Blood test*			
HbA1c (%)	Continuous		1
BNP (pg/mL)	Continuous		1
eGFR (ml/min/1.73 m^2^)	Continuous		1
PSA (ng/ml)	Continuous		1
Total		21	25
(b)			
*Demographic*			
Age	Continuous	1	1
BMI	Continuous	1	1
Delivery	Dichotomous	1	1
Menopause	Dichotomous	1	1
*Health behavior*			
Smoking status	Dichotomous	1	1
Alcohol habit	Dichotomous	1	1
Walking habit	Dichotomous	1	1
*Comorbidity*			
Hypertension	Dichotomous	1	1
Hyperlipidemia	Dichotomous	1	1
Diabetes	Dichotomous	1	1
Ischemic heart disease	Dichotomous	1	1
Stroke	Dichotomous	1	1
Kidney disease	Dichotomous	1	1
Cancer	Dichotomous	1	1
Depression	Dichotomous	1	1
Sleep disturbance	Dichotomous	1	1
Obstructive sleep apnea	Dichotomous	1	1
*OABSS*			
Question 1	Continuous	1	1
Question 2	Continuous	1	1
Question 3	Continuous	1	1
Question 4	Continuous	1	1
*Blood test*			
HbA1c (%)	Continuous		1
BNP (pg/mL)	Continuous		1
eGFR (ml/min/1.73 m^2^)	Continuous		1
Total		21	24

A total of 21 and 25 parameters of variables will be included in Model 1 and Model 2 for male, and 21 and 24 parameters will be included in Model 1 and Model 2 for female.

### Data cleaning

We will create frequency tables for categorical variables and box plots for the continuous variables. We will identify values out of plausible range (i.e. values that are clearly erroneous), and we will classify them as missing data. We will exclude some categorical predictors with very small prevalence. Continuous variables will be standardized and categorical variables will be transformed into dummy variables.

### Missing data

We will create 10 multiply imputed datasets using chained equations [19]. Each completed data set will be analyzed separately and the results will be combined by Rubin’s rules to account for imputation uncertainty [20].

### Model development

Logistic regression model will be used to develop Model 1 and Model 2 to predict a binary outcome, new-onset OAB. To avoid overfitting of data, we will employ a shrinkage method (LASSO) [21]. To find the optimal hyperparameter (λ) of penalization, a tenfold cross-validation will be performed.

### Model performance

We will evaluate the predictive accuracy of each model by R^2^ statistic. Model discrimination, i.e. the ability to classify the participants into high-risk or low-risk, will be evaluated using the C-statistic. Model calibration, agreement between observed outcomes and predictions, will be evaluated with calibration plots. To evaluate and compare the net benefit between models, decision curve analysis (DCA) will be performed [22].

### Model validation

We will use internal validation and temporal validation to evaluate the model performance [23]. Internal validation will be performed via bootstrap procedure repeated 200 times to calculate optimism-corrected R^2^, c-statistics and calibration slope. Temporal validity will be assessed by splitting the sample into 3 sets according to the year of baseline assessment (i.e. 2008, 2009 and 2010). We will use the first 2 sets (2008 and 2009) as the training set, and the 2010 set as the testing set, to evaluate discrimination and calibration.

### Statistical software

We will use R version 4.0.2 for our analyses. We will program a Shiny application in R to present the prediction results interactively.

## Discussion

We have described the protocol for developing a prediction model for OAB. To our knowledge, this is the first model to predict new-onset OAB based on a large-scale prospective cohort in the general population setting. Our prediction models have a large sample size and will incorporate various predictive variables based on previous studies and expert opinions. Moreover, we will develop a user-friendly web-based application to visualize the results of the prediction model. This may be very useful not only to healthcare providers but also to the general population, in interpreting and understanding the results. If we can develop an accurate prediction model for OAB and make it widely available through a web app, we will be able to detect high risk populations and thus intervene at an early stage, which may improve individual HRQOL and decrease the societal health care expenditure.

There are some limitations in this study. First, there may be a selection bias in the sample because the study participants were recruited not by random sampling but on a voluntary basis. However, compared with the previous study using randomly sampled Japanese population [24], Nagahama cohort showed similar prevalence of OAB [4], which may indicate absence of potential selection bias. Second, we will not be able to perform an external validation using an independent cohort, therefore we will not evaluate the general applicability of the models. Future studies will be necessary to demonstrate the external validity of the models with another cohort data.

As a future perspective, prediction models of incident OAB will need to be externally validated and there should be an investigation of their impact in clinical practice [25]. Our models will be developed by general population data and predictors of Model1 will include only self-reported questionnaires. This study aims to develop a model that is easy to use in the general population setting, and thus easy validate externally.

## Data Availability

It is not possible to share research data publicly because individual privacy could be compromised.

## Abbreviations

BMI: Body mass index
BNP: B-type natriuretic peptide
DCA: Decision curve analysis
eGFR: The estimated glomerular filtration rate
HRQOL: Health-related quality of life
OAB: Overactive bladder
OABSS: OAB symptom score
OSA: Obstructive sleep apnea
PCa: Prostate cancer
PSA: Prostate specific antigen
TRIPOD: Transparent Reporting of a Multivariable Prediction Model for Individual Prognosis or Diagnosis
